# Taurine chloramine decreases cell viability and cytokine production in blood and spleen lymphocytes from septic rats submitted to sepsis

**DOI:** 10.1186/cc12975

**Published:** 2013-11-05

**Authors:** Dhébora Mozena Dall'Igna, Jaqueline Maffezzolli da Luz, Francieli Vuolo, Fábio Klamt, Felipe Dal Pizzol

**Affiliations:** 1 Instituto Nacional de Ciência e Tecnologia Translacional em Medicina, Unidade Acadêmica de Ciências da Saúde, Universidade do Extremo Sul Catarinense, Criciúma, SC, Brazil; 2 Instituto de Ciências Básicas da Saúde, Universidade Federal do Rio Grande do Sul, Porto Alegre, RS, Brazil

## Introduction

Attention has been paid in recent years to studies showing immune cell death mechanisms during the course of sepsis in response to proinflammatory and anti-inflammatory mediators that are involved in its pathophysiology. Taurine (Tau) is an abundant amino acid in polymorphonuclear leucocytes that reacts with hypochlorous acid to form taurine chloramine (TauCl) under inflammatory conditions. In this context, we investigated potential interactions between lymphocytes and TauCl in rats submitted to cecal ligation and perforation (CLP), analyzing cell viability and cytokine secretion profile (TNFα, IFNγ, IL-6, IL-17A, IL-23 and IL-10).

## Materials and methods

Adult male rats were divided in two groups: sham and CLP that were killed 24 or 120 hours after sepsis induction to isolate lymphocytes from the blood and spleen. Lymphocytes (>95.0% purity determined by differentiation with Giemsa staining) were cultured for 24 hours at a concentration of 1 × 10^6 ^cells/ml and activated by 2 mg/ml concanavalin A. After 24 hours, Tau and TauCl were added at concentrations of 0.1, 0.2, 0.3, 0.4 and 0.5 mM for 1 hour. After this time, cells were incubated with MTT (500 μg/ml) for 3 hours to evaluate cell viability and supernatants were used to determine cytokine concentrations.

## Results

**Figure 1:**
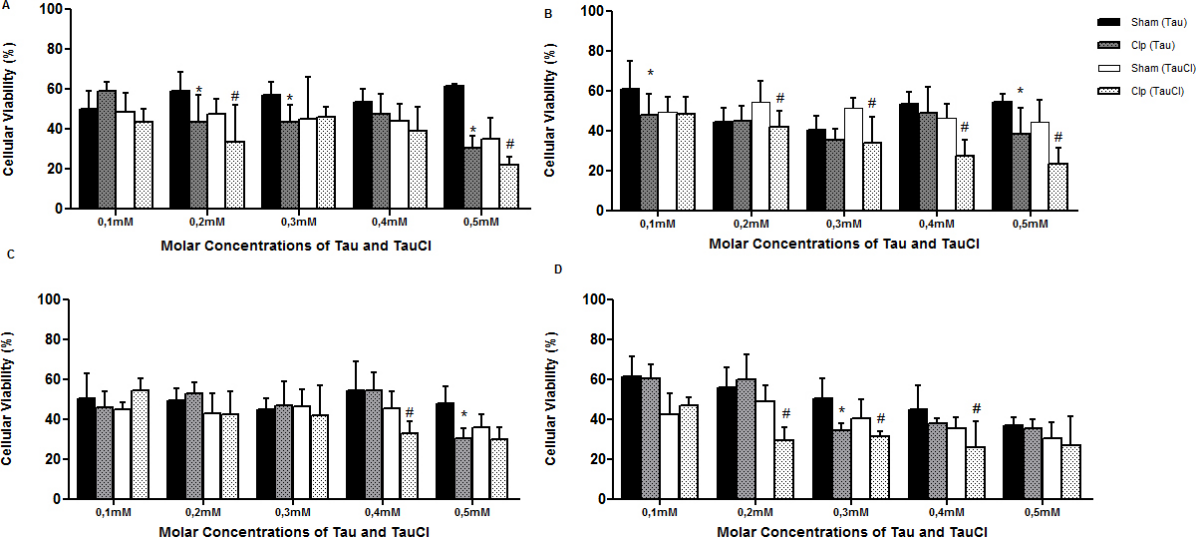
**Cell viability by MTT assay**. Viability of lymphocytes treated with Tau and TauCl in different molar concentrations. Rats were submitted to CLP or Sham, and 24 or 120 hours after the surgery their blood and spleens were collected, the lymphocytes were isolated, cultured and the cell viability was measured by MTT assay. **(A) **blood, 24 hours; **(B) **blood, 120 hours; **(C) **spleen, 24 hours; **(D) **spleen, 120 hours. *P < 0.05, compared with sham group (Tau-treated); #*P *< 0.05, compared with sham group (TauCl-treated), *n *= 5.

**Figure 2:**
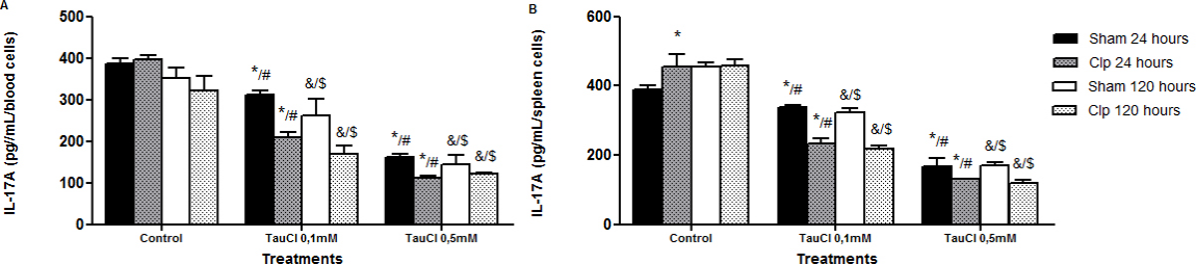
**Cytokine secretion**. Effect of TauCl on production of proinflammatory mediator IL-17A by Th17 lymphocytes. Activated lymphocytes (1 × 10^6 ^cells/ml) were preincubated with TauCl (0.1 or 0.5 mM) for 1 hour. After this, supernatants were collected and IL-17A was measured by ELISA. **(A) **blood; **(B) **spleen. Results are expressed as means ± SD. *Compared with sham control 24 hours; ^#^compared with Clp control 24 hours; ^&^compared with sham control 120 hours; ^$^compared with Clp control 120 hours, all with *P *< 0.05 significant (*n *= 5).

**Figure 3:**
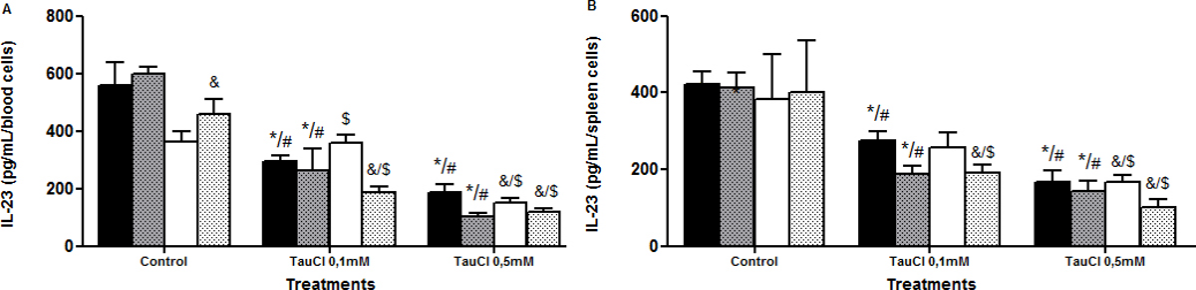
**Effect of TauCl on production of proinflammatory mediator IL-23 by Th17 lymphocytes**. Activated lymphocytes (1 × 10^6 ^cells/ml) were preincubated with TauCl (0.1 or 0.5 mM) for 1 hour. After this, supernatants were collected and IL-23 was measured by ELISA. **(A) **blood; **(B) **spleen. Results are expressed as means ± SD. *Compared with sham control 24 hours; ^#^compared with Clp control 24 hours; ^&^compared with sham control 120 hours; ^$^compared with Clp control 120 hours, all with *P *< 0.05 significant (*n *= 5).

**Figure 4:**
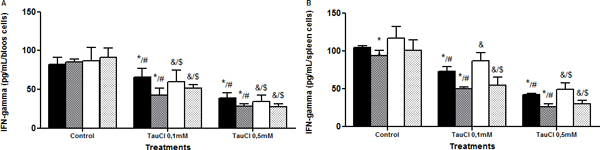
**Effect of TauCl on production of proinflammatory mediator IFNγ by Th1 lymphocytes**. Activated lymphocytes (1 × 10^6 ^cells/ml) were preincubated with TauCl (0.1 or 0.5 mM) for 1 hour. After this, supernatants were collected and IFNγ was measured by ELISA. **(A) **blood; **(B) **spleen. Results are expressed as means ± SD. *Compared with sham control 24 hours; ^#^compared with Clp control 24 hours; ^&^compared with sham control 120 hours; ^$^compared with Clp control 120 hours, all with *P *< 0.05 significant (*n *= 5).

**Figure 5:**
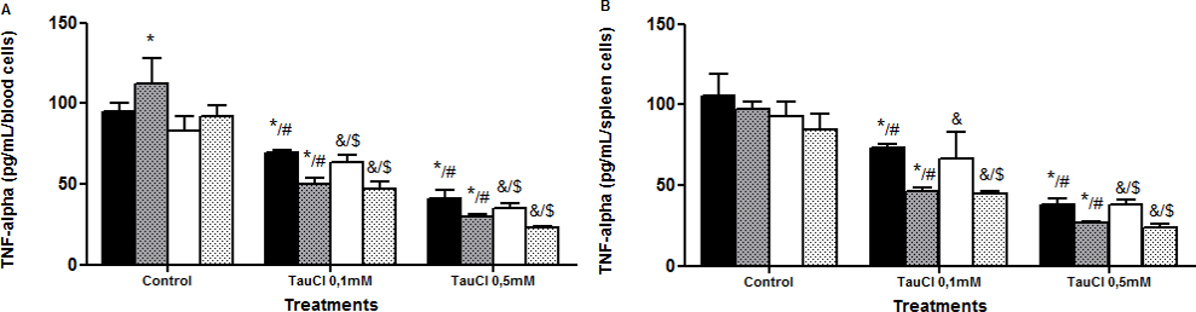
**Effect of TauCl on production of proinflammatory mediator TNFα by Th1 lymphocytes**. Activated lymphocytes (1 × 10^6 ^cells/ml) were preincubated with TauCl (0.1 or 0.5 mM) for 1 hour. After this, supernatants were collected and TNFα was measured by ELISA. **(A) **blood; **(B) **spleen. Results are expressed as means ± SD. *Compared with sham control 24 hours; ^#^compared with Clp control 24 hours; ^&^compared with sham control 120 hours; ^$^compared with Clp control 120 hours, all with *P *< 0.05 significant (*n *= 5).

**Figure 6:**
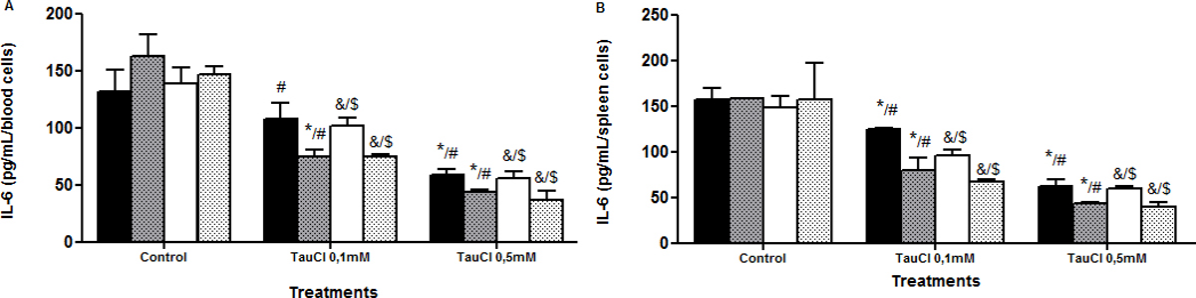
**Effect of TauCl on production of proinflammatory mediator IL-6 by Th2 lymphocytes**. Activated lymphocytes (1 × 10^6 ^cells/ml) were preincubated with TauCl (0.1 or 0.5 mM) for 1 hour. After this, supernatants were collected and IL-6 was measured by ELISA. **(A) **blood; **(B) **spleen. Results are expressed as means ± SD. *Compared with sham control 24 hours; ^#^compared with Clp control 24 hours; ^&^compared with sham control 120 hours; ^$^compared with Clp control 120 hours, all with *P *< 0.05 significant (*n *= 5).

**Figure 7:**
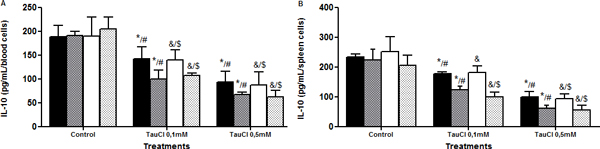
**Effect of TauCl on production of anti-inflammatory mediator IL-10 by Th2 lymphocytes**. Activated lymphocytes (1 × 10^6 ^cells/ml) were preincubated with TauCl (0.1 or 0.5 mM) for 1 hour. After this, supernatants were collected and IL-10 was measured by ELISA. **(A) **blood; **(B) **spleen. Results are expressed as means ± SD. *Compared with sham control 24 hours; ^#^compared with Clp control 24 hours; ^&^compared with sham control 120 hours; ^$^compared with Clp control 120 hours, all with *P *< 0.05 significant (*n *= 5).

Tau-treated cells exhibited better viability than those treated with TauCl, in both time and organs. TauCl, in a time and dose-dependent ratio, decreased cytokines secretion when compared with untreated cells. See Figures 1 to 7.

## Conclusion

These findings show a possible impairment in lymphocyte function promoted by TauCl, correlated with immunosuppression and cell death characteristic of the late stages of sepsis.

